# Repressor element-1 silencing transcription factor/neuronal restrictive silencer factor (REST/NRSF) can regulate HSV-1 immediate-early transcription via histone modification

**DOI:** 10.1186/1743-422X-4-56

**Published:** 2007-06-07

**Authors:** Rajeswara C Pinnoji, Gautam R Bedadala, Beena George, Thomas C Holland, James M Hill, Shao-chung V Hsia

**Affiliations:** 1 Department of Basic Pharmaceutical Sciences, College of Pharmacy, The University of Louisiana at Monroe, 700 University Avenue, Monroe, LA 71209 USA; 2 Department of Immunology and Microbiology, School of Medicine, Wayne State University, 540 East Canfield Avenue, Detroit, MI 48201 USA; 3 Department of Ophthalmology, Neuroscience, Pharmacology, and Microbiology LSU Eye Center and LSU Health Sciences Center, New Orleans, LA 70118 USA

## Abstract

**Background:**

During primary infection of its human host, Herpes Simplex Virus Type-1 (HSV-1) establishes latency in neurons where the viral genome is maintained in a circular form associated with nucleosomes in a chromatin configration. During latency, most viral genes are silenced, although the molecular mechanisms responsible for this are unclear. We hypothesized that neuronal factors repress HSV-1 gene expression during latency. A search of the HSV-1 DNA sequence for potential regulatory elements identified a Repressor Element-1/Neuronal Restrictive Silencer Element (RE-1/NRSE) located between HSV-1 genes ICP22 and ICP4. We predicted that the Repressor Element Silencing Transcription Factor/Neuronal Restrictive Silencer Factor (REST/NRSF) regulates expression of ICP22 and ICP4.

**Results:**

Transient cotransfection indicated that REST/NRSF inhibited the activity of both promoters. In contrast, cotransfection of a mutant form of REST/NRSF encoding only the DNA-binding domain of the protein resulted in less inhibition. Stably transformed cell lines containing episomal reporter plasmids with a chromatin structure showed that REST/NRSF specifically inhibited the ICP4 promoter, but not the ICP22 promoter. REST/NRSF inhibition of the ICP4 promoter was reversed by histone deacetylase (HDAC) inhibitor Trichostatin A (TSA). Additionally, chromatin immuno-precipitation (ChIP) assays indicated that the corepressor CoREST was recruited to the proximity of ICP4 promoter and that acetylation of histone H4 was reduced in the presence of REST/NRSF.

**Conclusion:**

Since the ICP4 protein is a key transactivator of HSV-1 lytic cycle genes, these results suggest that REST/NRSF may have an important role in the establishment and/or maintenance of HSV-1 gene silencing during latency by targeting ICP4 expression.

## Background

Lytic infection by Herpes Simplex Virus Type-1 (HSV-1) typically occurs in epithelial cells [1]. During these infections, HSV-1 expresses more than eighty genes in a sequential regulatory cascade [2]. Immediate-early (IE or α) gene products are the first group to be transcribed followed by early (E or β) and late (L or γ) gene expressions [3]. Expression of E and L genes depends on the availability of IE proteins, thus demonstrating their importance in the lytic cycle. During lytic infection, viral DNA is not associated with nucleosomes. HSV-1, like other alphaviruses, also establishes latent infections in sensory neurons of the peripheral nervous system [4,5]. In contrast to lytic infection, latency is distinguished by the absence of viral polypeptides and a highly restricted pattern of transcription [2,6]. Studies of HSV-1 latency in animal models have indicated that the majority of viral DNA is maintained in a circular form and associated with nucleosomes in a regularly spaced chromatin pattern [7]. However, detailed studies of latent viral chromatin have been difficult to conduct and the role of chromatin in viral latency remains to be defined.

We hypothesize that a repressive chromatin structure and specific neuronal transcription factors contribute to transcription inhibition during latency. We identified a Restrictive Element-1/Neuronal Restrictive Silencer Element (RE-1/NRSE) located between the promoters for the Immediate-Early ICP4 and ICP22 genes. RE-1/NRSE is the binding site of RE1-Silencing Transcription factor/Neuronal Restrictive Silencer Factor (REST/NRSF) [8]. REST/NRSF is a zinc finger transcription factor originally defined as a silencer protein for the neuron-specific gene SCG10 [9]. Recent studies revealed that REST/NRSF exhibits ubiquitous presence [10] and plays roles in neurogenesis, neural plasticity, tumor suppression, and cancer progression through transcription regulation [11]. REST/NRSF and its corepressor complex CoREST have not been linked to HSV-1 biology until recent studies showing that the HSV-1 IE protein ICP0 dissociates HDAC 1 and 2 from the REST/CoREST complex [12]. However, the putative role of REST/NRSF on HSV-1 transcription has not been elucidated.

We investigated the effect of REST/NRSF on HSV-1 IE transcription using an episomally replicating plasmid that associates with nucleosomes in a standard chromatin configuration in stably transfected cell lines [13]. Plasmids containing the secreted alkaline phosphatase (SEAP) reporter gene driven by either ICP4 or ICP22 promoter were characterized in transient transfections and in stably transformed cells. In transient transfections, REST/NRSF repressed the activity of both the ICP22 and ICP4 promoters. However, in stably transfected cells, REST/NRSF exhibited significant inhibition of the ICP4 promoter but only moderate reduction on ICP22 activity in chromatin context. The histone deacetylase inhibitor trichostatin A was sufficient to reverse the inhibition of ICP4 in stable cells, indicating the role of histone deacetylation in REST/NRSF mediated regulation in this system. ChIP assays revealed that CoREST was recruited to the proximity of HSV-1 RE-1/NRSE and that histone H4 acetylation was reduced in the presence of REST/NRSF. These results demonstrate the roles of REST/NRSF in the regulation of HSV-1 IE transcription.

## Results

### A putative RE-1/NRSE was found within the promoter of HSV-1 ICP22

**Figure 1:**
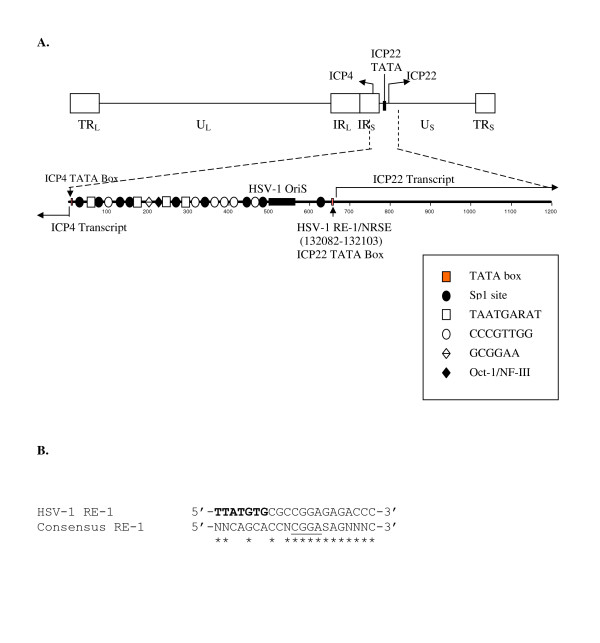
**HSV-1 genome and HSV-1 RE-1/NRSE sequence**. **A**. HSV-1 genome is composed of two covalently linked components, designated as U_L _(Unique Long) or U_S _(Unique Short). Each component contains unique sequences bracketed by inverted and terminal repeats (TR_L _and IR_L_). The ICP22 gene is present in U_S _and one of the two ICP4 is located at the junction of U_S _and IR_S _since the genes that are encoded within the repeat sequences are present twice in the viral genome. The HSV-1 RE-1/NRSE is mapped from 132082 to 132103 according to the HSV-1 complete genome sequence accession number X14112. **B**. Putative HSV-1 RE-1/NRSE. The HSV-1 RE-1/NRSE sequence was identified to overlap ICP22 TATA box (Bold) compared to consensus sequence. The core sequence is underlined. The matching result indicated that the homology is 76%. W: A or T; N: any nucleotide; S: G or C; Y: C or T; R: A or G.

We identified a putative HSV-1 RE-1/NRSE having 76% identity to the published consensus sequence [14] in the intergenic region between the ICP4 and ICP22 coding sequences (Fig. 1A). The location of various *cis-*acting elements was shown according to literature [15]. The HSV-1 RE-1 core sequence exhibited 100% identity to the consensus RE-1/NRSE. This HSV-1 RE-1/NRSE is located immediately downstream of the TATA box of the ICP22 promoter and 660 bp upstream of the ICP4 transcription initiation site (Fig. 1B).

### REST/NRSF repressed ICP22 and ICP4 promoter activity in transient co-transfection

**Figure 2:**
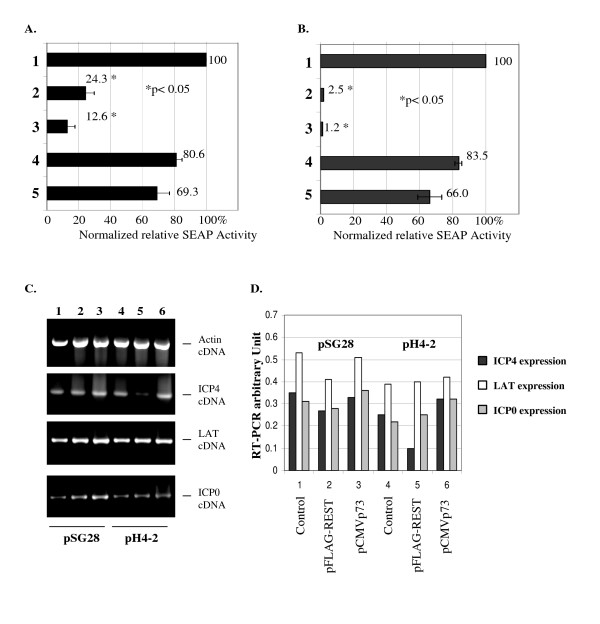
**REST/NRSF inhibits ICP22 and ICP4 promoter activity in transient cotransfection**. A. Cotransfection of pICP22 with different amount of expression plasmids was performed followed by SEAP assay to analyze the regulatory effect of REST/NRSF on ICP22 promoter. 1. pICP22 and control plasmid. 2. pICP22 and pFLAG-REST (1:1). 3. pICP22 and pFLAG-REST (1:2). 4. pICP22 and pCMVp73 (1:1). 5. pICP22 and pCMVp73 (1:2). The asterisk *P *values represent Student's *t *tests in pairwise comparisons to the Lane 1 pICP22 + Control plasmid. The error bars represent standard deviations. The data were calculated and graphed using Microsoft Excel. B. Cotransfection of pICP4 with different amount of expression plasmids was performed followed by SEAP assay to analyze the regulatory effect of REST/NRSF on ICP4 promoter. 1. pICP4 and control plasmid. 2. pICP4 and pFLAG-REST (1:1). 3. pICP4 and pFLAG-REST (1:2). 4. pICP4 and pCMVp73 (1:1). 5. pICP4 and pCMVp73 (1:2). The *P *values represent Student's *t *tests in pairwise comparisons to the Lane 1 pICP4 + Control plasmid. The error bars represent standard deviations. The data were calculated and graphed using Microsoft Excel. C. Plasmid pSG28 and pH4-2 were cotransfected with pFLAG-REST or pCMVp73 followed by RNA isolation and RT-PCR. 1. pSG28 and control plasmid (1:1). 2. pSG28 and pFLAG-REST (1:1). 3. pSG28 and pCMVp73 (1:1). 4. pH4-2 and control plasmid (1:1). 5. pH4-2 and pFLAG-REST (1:1). 6. pH4-2 and pCMVp73 (1:1). D. The RT-PCR results were quantified by Kodak Gel-Logic 100 system to measure the sum intensity of each band representing the regulatory effect of REST/NRSF on ICP4 transcription.

The regulatory effect of REST/NRSF on the ICP22 promoter was first measured by transient transfection assays. We performed cotransfection of pICP22 (containing the SEAP reporter gene under the control of the ICP22 promoter) and pFLAG-REST into 293HEK cells at the molar ratio of 1:1 or 1:2 (pICP22: pFLAG-REST). SEAP assays were performed three days post-transfection according to the manufacturer's protocols. The results indicated that the REST/NRSF repressed the ICP22 promoter activity to 23% and 9% of control levels at the ratio of 1:1 and 1:2, respectively (Fig. 2A). Similar co-transfections were done with pCMVp73, which expresses a truncated form of REST/NRSF containing only the protein's DNA binding domain. The ICP22 promoter retained 70–80% of its activity in the presence of REST/NRSF mutant p73 (Fig. 2A). Empty vector pREP-SEAP transfection was performed and exhibited very low basal activity (data not shown).

The regulatory effect of REST/NRSF on the ICP4 promoter was investigated by the same strategy. Cotransfection of pICP4 and pFLAG-REST at the molar ratio of 1:1 or 1:2 (pICP4: pFLAG-REST) revealed that REST/NRSF inhibited the ICP4 promoter activity to 2.5% and 1.2%, respectively, (Fig. 2B). The mutant vector pCMVp73 exerted only a weak repressive effect on the promoter (Fig. 2B). These results indicated that REST/NRSF inhibited ICP22 and ICP4 promoter activity in transient transfections.

To further confirm the regulatory effect of REST/NRSF on HSV-1 transcription, plasmid pSG28 and pH4-2 were cotransfected with pFLAG-REST or pCMVp73 to analyze the effect of HSV-1 RE-1/NRSE on the gene regulation of ICP4. It is noted that both pSG28 and pH4-2 contain the complete open reading frame of ICP4, LAT, and ICP0. The RT-PCR results indicated that REST/NRSF and mutant p73 exhibited no major effect on the ICP4 promoter of pSG28, which does not have HSV-1 RE-1/NRSE (Fig. 2C). However, the ICP4 promoter of pH4-2 (containing HSV-1 RE-1/NRSE) was significantly repressed by REST/NRSF but not p73, which showed no inhibition at all (Lane 4, 5, and 6, Fig. 2C). The quantification analysis revealed that 62% of ICP4 promoter activity in pH4-2 was repressed by REST/NRSF, compared to 28% in pSG28 (Fig. 2D; black bar, lane 4 and 5). In addition, REST/NRSF exhibited no repression on LAT and ICP0 transcription in both plasmids (Fig. 2C and 2D, white and gray bars). These results demonstrated that REST/NRSF required HSV-1 RE-1/NRSE to exert its regulatory effect on ICP4 promoter.

### Nucleosomes are associated with episomal plasmids in stable cells

**Figure 3:**
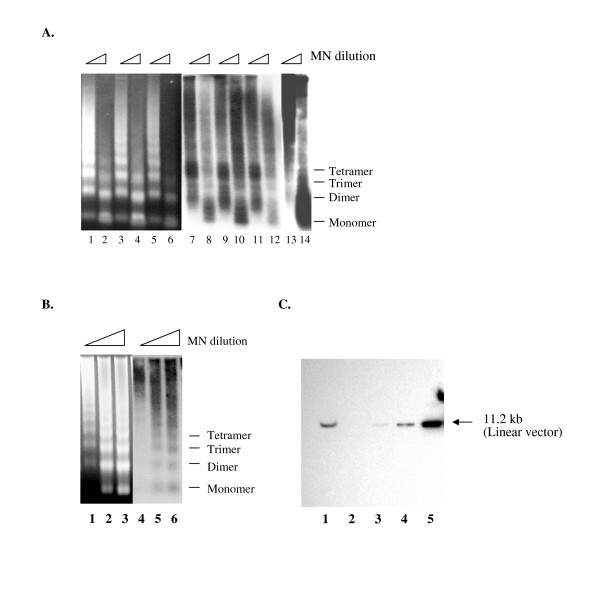
**Episomal plasmids remained in extra-chromosomal form and associated with nucleosomes in stable cells**. **A**. Analysis of nucleosomal formation on transient transfected reporter plasmid. Plasmid pICP4 was cotransfected with control vector (Lane 1, 2, 7, and 8), pFLAG-REST (Lane 3, 4, 9, and 10), and pCMVp73 (Lane 5, 6, 11, and 12). Lane 1–6: Ethidium bromide staining. Lane 7–12: plasmid-specific nucleosmal protected ladder detected by vector probe. Lane 13 and 14: Naked plasmid digestion control. **B**. Analysis of nucleosomal formation on episomal plasmids. The nuclei from stable 293HEK-pICP22 were subjected to MNase digestion followed by Southern blot hybridization. Lane 1–3: ethidium bromide staining of the genomic DNA nucleosomal protected ladder. Lane 4–6: episomal plasmid-specific nucleosmal protected ladder detected by vector probe. **C**. Examination of episomal status of plasmids in the stable cells harboring pICP4 by Southern hybridization. Lane 1. Total DNA purified from 1.28 × 10^6 ^stable cells; Lane 2: 10 pg plasmid; Lane 3: 0.1 ng plasmid DNA; 4: 1 ng plasmid DNA; 5: 10 ng plasmid DNA.

To assess the effect of REST/NRSF on nucleosomal formation during transient transfection, we performed MNase digestion on cells two days after the transfection of reporter plasmids and expression vectors followed by Southern blot hybridization using probes against the plasmid. The hybridization results revealed an irregular pattern of nucleosomal ladder compared to the genomic ladder and naked digestion control, suggesting that histones were associated with the plasmid but not in a *bona fide *nucleosomal structure (Fig. 3A). Expression of REST/NRSF or p73 did not affect the nucleosomal configuration.

To establish reporter plasmids that are assembled into chromatin, we subjected cells transfected with the episomally replicating pICP4 or pICP22 plasmids to hygromycin B selection. The stable cells containing pICP22 or pICP4 were established after 10 days of selection. To examine the chromatin structure of the episomal plasmids, nuclei from parental and stable cells were again subjected to different concentrations of MNase digestion followed by Southern blot hybridization. Ethidium bromide staining of the agarose gel revealed the nucleosome protected ladder characteristic of genomic DNA (Fig. 3B, Lane 1–3), indicating that the protocol of MNase digestion was effective. Southern hybridization showed a plasmid-specific nucleosome protected ladder resembling the genomic ladder (Fig. 3B, Lane 4 to 6). The nucleosomal ladder of Southern hybridization is not an artifact since the samples from parental cells exhibited no signal at all (data not shown). These results demonstrated that the plasmids are associated with nucleosomes in the stably transfected cells. To test for integration of plasmids in cells, total DNA purified from stable cells and plasmid DNA was digested with NcoI, which cuts the plasmid once, followed by Southern blot hybridization using vector probe. The results detected a single band with the size of 11.2 kb, equivalent to the size of the original plasmid (Fig. 3C). The results concluded that the plasmids remained in an extra-chromosomal form since integrated plasmid digestion would exhibit different sizes.

### REST/NRSF repressed ICP4 but not ICP22 promoter activity in stable cell lines

**Figure 4:**
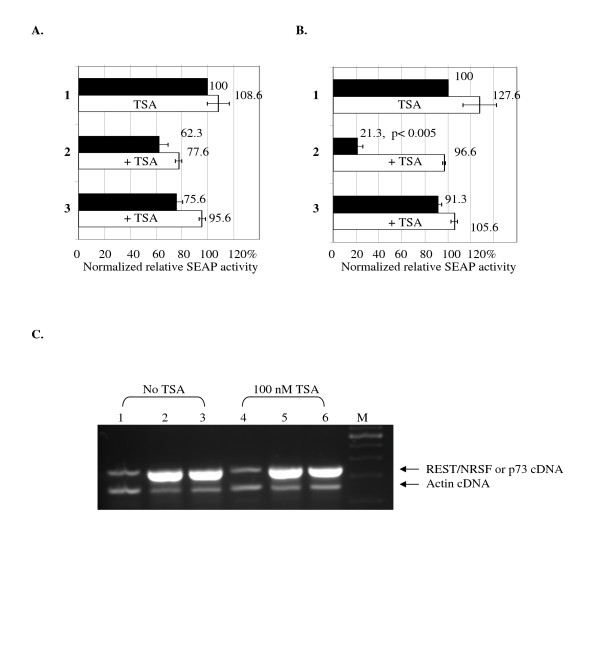
**REST/NRSF exhibited significant reduction on ICP4 promoter activity and the REST/NRSF-mediated repression was reversed by HDAC inhibitor TSA**. **A**. Stable 293HEK cells containing pICP22 (293HEK-pICP22) was transfected with control plasmid, pFLAG-REST, or pCMVp73 in the presence (white bar) or absence (black bar) of 100 nM TSA. 1. transfected with control plasmid. 2. transfected with 1 μg of pFLAG-REST. 3. transfected with 1 μg of pCMVp73. The *P *values represent Student's *t *tests in pairwise comparisons to the control without TSA. **B**. Stable 293HEK cells containing pICP4 (293HEK-pICP4) was transfected with control plasmid, pFLAG-REST, or pCMVp73 in the presence (white bar) or absence (black bar) of 100 nM TSA. 1. transfected with control plasmid. 2. transfected with 1 μg of pFLAG-REST. 3. transfected with 1 μg of pCMVp73. The *P *values represent Student's *t *tests in pairwise comparisons to the control without TSA. **C**. Effect of TSA on REST/NRSF and p73 transcriptions in 293HEK-pICP4. M: 100 bp ladder. 1. Transfected with 1 μg control plasmid. 2. Transfected with 1 μg pFLAG-REST. 3. Transfected with 1 μg pCMVp73. 4. Transfected with 1 μg control plasmid with 100 nM TSA. 5. Transfected with 1 μg pFLAG-REST with 100 nM TSA. 6. Transfected with 1 μg pCMVp73 with 100 nM TSA. The cDNA from REST/NRSF and actin were marked by arrows.

To study the regulatory effect of REST/NRSF in a chromatin context, we transfected stable cells harboring pICP22 (293HEK-pICP22) with pFLAG-REST or pCMVp73. The cells were harvested for SEAP assays 72 hours after transfection. These assays showed that the FLAG-REST and p73 proteins exerted only minor inhibitory effects on the ICP22 promoter in 293HEK-pICP22 cells (Fig. 4A). Promoter activity was mildly reduced to 63% and 78% of control levels, respectively, by these proteins.

In contrast, we observed a significant inhibitory effect on the ICP4 promoter by REST/NRSF in stable cells harboring pICP4 (293HEK-pICP4). SEAP assays showed that ICP4 promoter activity was reduced to 21% of control levels by REST/NRSF. On the other hand, ICP4 promoter activity was essentially unchanged (94%) by the mutant p73 (Fig. 4B). These results suggested that the REST/NRSF may cooperate with chromatin to inhibit ICP4 transcription and the C-terminus of REST/NRSF played critical roles in this directional repression.

### The REST/NRSF-mediated ICP4 inhibition was released by HDAC inhibitor TSA

We predicted that the directional repression of ICP4 by REST/NRSF is through histone deacetylation since the C-terminal part of REST/NRSF was reported to recruit HDAC [11,16]. To assess the role of histone deacetylation in this system, 293HEK-pICP22 and 293HEK-pICP4 stable cell lines were transfected with the control plasmid, pFLAG-REST, or pCMVp73 with or without TSA. The HDAC inhibitor TSA (100 nM, Sigma, MO) was added to the media 24-hour after transfection. Cells were harvested for SEAP assays 72-hour after transfection. In 293HEK-pICP22 cells transfected with pFLAG-REST, very little effect (1.2-fold induction) was observed in the presence of TSA (Fig. 4A). However, in 293HEK-pICP4 cells transfected with pFLAG-REST, TSA treatment increased ICP4 promoter activity 4.8-fold (Fig. 4B). We observed that the ICP4 promoter activity was not affected by TSA in the absence of REST/NRSF (Fig. 4B). To confirm that TSA did not affect the expression of REST/NRSF, we isolated total RNA from the transfected cells and performed RT-PCR using primers against REST/NRSF and actin. The data revealed that the transcriptions of REST/NRSF and p73 remained in the same pattern, indicating that the reactivation of ICP4 transcription by TSA is not due to the reduction of REST/NRSF expression (Fig. 4C). These results indicated that histone acetylation has a critical role in ICP4 gene expression in this system and that REST/NRSF may induce histone deacetylation to repress transcription of ICP4 in the context of chromatin.

### REST/NRSF interacted with HSV-1 RE-1/NRSE

**Figure 5:**
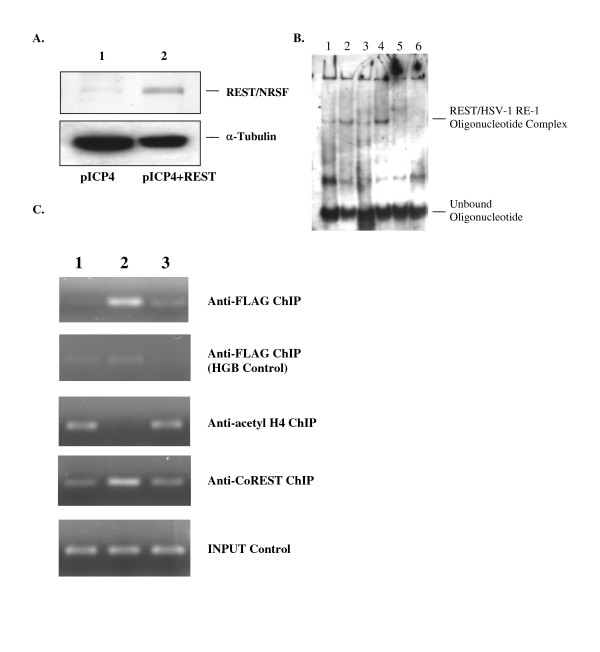
**REST/NRSF recruited corepressor CoREST to HSV-1 RE-1/NRSE and induced histone H4 deacetylation**. **A**. Over-expression of REST/NRSF in 293HEK cells by transfection of pFLAG-REST. Lane 1: 293HEK cells transfected with pICP4. Lane 2: 293HEK cells transfected with pICP4 and pFLAG-REST. Immunoblot was performed using anti-REST antibody. **B**. EMSA using transfected cell extract. Lane 1. Labeled HSV-1 RE-1 ds oligo incubated with extract isolated from cells transfected with control plasmid. Lane 2. Labeled HSV-1 RE-1 ds oligo incubated with extract isolated from cells transfected with pFLAG-REST. Lane 3: Labeled mutant oligo incubated with extract isolated from cells transfected with pFLAG-REST. Lane 4: Labeled HSV-1 RE-1 ds oligo incubated with extract isolated from cells transfected with pCMVp73. Lane 5: Labeled wild-type ds oligo containing 10× unlabeled wild-type oligo incubated with extract isolated from cells transfected with pFLAG-REST. Lane 6: Labeled wild-type ds oligo containing 25× unlabeled wild-type oligo incubated with extract isolated from cells transfected with pFLAG-REST. Noted that endogenous REST/NRSF produced a shifted band (Lane 1). **C**. Analysis of REST/NRSF binding, histone H4 acetylation alteration, and CoREST recruitment by ChIP. 293HEK-pICP4 cell line was transfected with pFLAG-REST and subjected to ChIP assay. Samples prior to the immuno-precipitation are used for input control. The samples were amplified by PCR and subjected to 1.2% agarose electrophoresis staining with ethidium bromide. 1. pICP4 + Control. 2. pICP4 + pFLAG-REST. 3. pICP4 + pCMVp73.

To confirm the expression of the fusion protein FLAG-REST in the transfected 293HEK cells, we performed Western Blotting using the anti-REST antibody. The results indicated that the cells transfected with 1 μg of pFLAG-REST showed significant increase of REST/NRSF expression compared to the control (Fig. 5A). The anti-REST antibody (produced against amino acid residues 801–1097 of human REST/NRSF) did not recognize mutant p73 (amino acids 73–545).

To demonstrate the *in vitro *binding of REST/NRSF to the HSV-1 RE-1/NRSE, we carried out EMSA using DIG-11-ddUTP-labeled wild-type ds oligonucleotide (oligo) containing HSV-1 RE-1/NRSE and oligo with core sequence mutation. Extract isolated from cells transfected with pFLAG-REST, pCMVp73, or control plasmids were used for *in vitro *interaction. The results revealed that both REST/NRSF and mutant p73 yield strong, increased signal of shifted bands while wild type oligo was used, demonstrating that REST/NRSF and its DNA binding domain bound to HSV-1 RE-1/NRSE *in vitro *(Fig. 5B, compare lane 1 to 2 and 4). The mutant oligo showed no band shifting, indicating that core sequence of HSV-1 RE-1/NRSE is critical for the interaction (Fig. 5B, lane 3). The competition analysis using unlabeled wild-type oligo abolished the shifted bands, indicating that the interaction is specific (Fig. 5B, lane 5 and 6).

To investigate the *in vivo *binding of REST/NRSF to the HSV-1 RE-1/NRSE, we performed ChIP using anti-FLAG^® ^M2 Affinity Gel. The results showed strong PCR signal from pFLAG-REST IP sample compared to the control and pCMVp73, indicating that FLAG-tagged REST/NRSF was recruited to the minichromosomes (Fig. 5C, anti-FLAG ChIP). The interaction of mutant p73 was not detected due to the lack of FLAG-tag. In addition, only a very weak signal was detected using primers against hygromycin B resistance gene (Fig. 5C, HGB control), indicating that the binding of REST/NRSF was specific to the promoter region and the shearing of minichromosome was sufficient. These results indicated that REST/NRSF and mutant p73 bound to the HSV-1 RE-1/NRSE.

### REST/NRSF reduced the acetylation of histone H4 and CoREST was recruited to the proximity of the ICP4 promoter

To analyze the participation of corepressor to HSV-1 RE-1/NRSE, we performed ChIP using the anti-CoREST antibody. The results showed a much stronger signal from the FLAG-REST transfected samples, indicating that CoREST is recruited to HSV-1 RE-1/NRSE through REST/NRSF (Fig. 5C, anti-CoREST ChIP). We further investigated the histone acetylation status by the same method using antibody against acetylated histone H4. The results revealed that acetylation was reduced in the presence of REST/NRSF compared to the control and p73 (Fig. 5C). These results indicated that CoREST is recruited to the HSV-1 RE-1/NRSE via the interaction of REST/NRSF in a chromatin context and this interaction reduced the histone acetylation of histone H4 in the proximity of HSV-1 IE promoters.

## Discussion

In this study, we identified a RE-1/NRSE site in the HSV-1 genome between the ICP4 and ICP22 Immediate Early promoters and showed that REST/NRSF exerted a chromatin state-dependant repressive effect on the activity of these promoters. In stably transformed cells, the plasmids pICP4 and pICP22, respectively containing the HSV-1 ICP4 and ICP22 promoters and the SEAP reporter gene, associated with nucleosomes in a regular chromatin array. Thus the chromatin structure of these promoters should resemble their structure in latently infected cells more closely than in any other available model system. Transfection of pFLAG-REST into these cells resulted in a substantial decrease in ICP4 promoter activity. This effect required the effector domain of REST/NRSF since pCMVp73, which contains only the DNA-binding domain, had little effect on the ICP4 promoter. Repression of the ICP4 promoter by REST/NRSF was reversed by Trichostatin A, suggesting that it was mediated, at least in part, by histone deacetylation. This was confirmed by ChIP analysis, which showed a significant reduction in the amount of acetylated histone H4 associated with the ICP4 promoter in pFLAG-REST transfected cells. Consistent with this, ChIP analysis also showed that CoREST, which is able to recruit histone deacetylases, was also associated with the ICP4 promoter in pFLAG-REST transfected cells. This was further supported by the p73 data, which indicated that the REST/NRSF mutant lacking effector region does not recruit CoREST to the promoter and failed to deacetylate histone H4 at the promoter. In contrast to the ICP4 promoter, the ICP22 promoter was relatively insensitive to repression by REST/NRSF. Since the RE-1/NRSE is located approximately 660 bp upstream of the ICP4 promoter but is adjacent to the ICP22 TATA box, this may be due to directional or distance-dependent effects, or it may indicate that REST/NRSF is able to exert promoter-specific effects in a chromatin context.

In transiently transfected cells, the transfected plasmids do not associate with nucleosomes in a regular chromatin pattern and thus resemble viral DNA in lytically infected cells. REST/NRSF repressed both the ICP4 and ICP22 promoter in these cells. In these conditions, repression of transcription from the ICP22 promoter might be due to steric hindrance of the TATA box and/or the transcription initiation site. However, repression of the ICP4 promoter must be due to other repressive effects of REST/NRSF.

ICP22 and ICP4 have fundamentally different roles in HSV-1 replication. ICP4 is essential for expression of E and L genes [17]. ICP22 is not essential, but is required for efficient replication [18] and transcription of HSV in certain cell types [19]. It is needed for maximal expression of the γ1 and γ2 genes, probably due to the fact that ICP22 induces phosphorylation on the large subunit of cellular RNA polymerase II [20]. The role of ICP22 in HSV-1 latency is not fully understood. No direct role for ICP22 in establishment or maintenance of latent infection has been demonstrated. However, given the transactivating effect of ICP4, it may be more important for the virus to effectively repress this promoter during these phases of latency, and this may be mediated by REST/NRSF.

This study complements a recent report showing that CoREST, a component of corepressor complex REST/CoREST/HDACs, exhibits a sequence similarity at the amino terminus to HSV-1 IE protein ICP0, and that the HDACs may be dissociated from the corepressor complex by ICP0 in cells infected by wild-type viruses [12]. Thus, the authors predicted that the REST/CoREST/HDACs complex could cause HSV-1 gene silencing at low multiplicities of infection (MOI). Our results revealed that CoREST is recruited to HSV-1 RE-1/NRSE in the presence of REST/NRSF, supporting the hypothesis that REST/CoREST complexes participated in the regulatory effect on HSV-1 IE genes. We predict this mechanism applies to HSV-1 latent infection since REST/NRSF is present in neurons [10,21]. This is supported by studies showing that HSV mutants lacking functional ICP0 exhibit poor reactivation efficiency [22-24]. Furthermore, at low MOI, nonneuronal cells infected with ICP0 deletion mutants produce about 100-fold less virus compared to cells infected with wild-type virus. In these experiments, the lack of ICP0 may have resulted in the failure to disrupt the inhibitory effect of the REST/CoREST/HDACs complex on IE genes. Our results showed that REST/NRSF inhibited ICP4 promoter activity in a chromatin context, suggesting REST/NRSF and repressive chromatin could maintain gene silencing during the establishment of a transcriptionally silent state and could provide a possible mechanism for long-term persistence through histone modification. Histone modification is suggested to regulate transcription through a mechanism of "histone code"[25]. Our ChIP analysis indicated that at least one type of histone modification (acetylation of histone H4) participated in the gene regulation. It is not clear why REST/NRSF failed to repress the ICP22 promoter activity in a chromatin context. However, other nearby *cis*-acting elements may modulate the effects of REST/NRSF. Sequence analysis indicates that the OriS, located between the ICP4 and ICP22 promoters, may fold into a stable hairpin [26]. A recent study suggested that the OriS stem-loop structure encodes a microRNA that may regulate HSV and host gene expression [27]. Our hypothesis is that a chromatin insulator-like element participated in the regulation of ICP22. Another recent study identified a cluster of CTCF motifs, designated CTRS3, in the intron of the ICP22 gene [28]. CTCF elements can function as insulators shielding genes in one region of a chromosome from the regulatory effects of another region [29]. Two classes of insulators have been reported, enhancer-blocking and boundary/barrier elements [28]. The former impedes the enhancer function and the latter prevents the spreading of repressive heterochromatin into the transcriptionally active area. Based on our results, we predict that CTRS3 and/or OriS act as a boundary/barrier element to prevent the repressive chromatin from spreading to the ICP22 promoter and thus aborted the inhibitory effect of REST/NRSF on ICP22 in the presence of nucleosomes. Further studies using neuronal models and promoter deletion are required to elucidate the complex regulatory mechanisms.

## Conclusion

**Figure 6:**
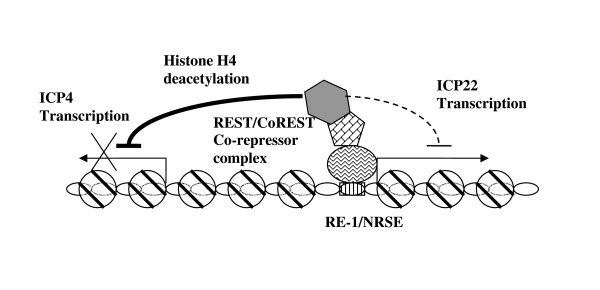
**Proposed model of HSV-1 ICP4 and ICP22 regulation by REST/NRSF**. REST/NRSF interacts with HSV-1 RE-1 in chromatin and represses ICP4 by recruiting CoREST and HDAC to induce hypoacetylation around the ICP4 promoter.

In summary, we have provided the first direct evidence indicating that REST/NRSF can regulate HSV-1 IE gene expression. We propose that during the establishment and maintenance of latent infection, REST/NRSF binds to the HSV-1 RE-1/NRSE in a chromatin context and recruits CoREST/HDAC complexes. As a result, the repressor complexes inhibit the ICP4 transcription and produce long-term repression via histone deacetylation, and possibly chromatin methylation (Fig. 6). More experiments are underway to investigate the role of REST/NRSF on HSV-1 gene regulation using neuronal cells and animal models.

## Methods

### Construction of plasmids and PCR amplification

The construction of episomal vector pICP4 and pICP22 were essentially described previously [30]. REST/NRSF expression vectors pFLAG-REST and pCMVp73 are gifts from Dr. Gail Mandel (SUNY, Stony Brook, NY). Plasmid pFLAG-REST expresses fusion protein REST/NRSF with a FLAG tag and pCMVp73 is a mutant REST/NRSF encoding 485 amino acids of the DNA-binding domain [31].

The plasmid pH4-2, based on pUC19, contains the Hind III restriction fragment (non-prototype structure) covering the entire long and short internal repeats. Plasmid pSG28, based on pUC18, contains EcoR I fragment covering HSV-1 sequence from 106785 to 131534. Both plasmids contain open reading frame of ICP4, LAT, and ICP0. However, pH4-2 contains HSV-1 RE-1/NRSE and ICP22 promoter but pSG28 does not. Plasmid pGL3-basic (Promega, Madison, WI) was used as control for transfection.

### Cell culture, transfection and selection for stable cell lines

The 293HEK cell is a human embryonic kidney cell line and was purchased from the American Type Culture Collection (ATCC) and maintained in DMEM medium supplemented with 10% fetal bovine serum (FBS). For transfection, cultures of cells were prepared for transfection by plating 5 × 10^5 ^cells in 60 mm culture dishes. After overnight incubation, the cells were transfected with 5 μg plasmid DNA complexed with 20 μl Superfect reagent (Qiagen, Valencia, CA) according to the procedures recommended by the manufacturer. To obtain stably transformed cell lines, the transfected cells were trypsinized 72 hours after transfection and replated in T25 flasks in medium containing 200 μg/ml of hygromycin B (Invitrogen, CA).

### Western Blot analysis

293HEK cells (2 × 10 ^6^) were transfected with pFLAG-REST or pCMVp73. For the preparation of cell extracts, the monolayers were washed with ice-cold phosphate-buffered saline (PBS), and the cells were lysed by adding 5 ml of cell extract buffer (25 mM Tris-HCl, 50 mM β-glycerol phosphate, 1 mM EGTA, 0.5 mM EDTA, 5% glycerol, 1% Triton X-100, 0.1 mM Benzamidine, 0.5 M Na_3_VaO_4_, 0.5 M phenylmethylsulfonyl fluoride, and protease inhibitors cocktail tablets from Roche). Protein concentration was determined by the Bradford protein assay (Bio-Rad, Hercules, CA). Proteins were subjected to 10% sodium dodecyl sulfate polyacrylamide gel electrophoresis and transfered onto nitrocellulose membranes. The blots were blocked using PBS with 5% (wt/vol) non-fat dry milk and washed in PBS. Anti-REST rabbit polyclonal antibody (Upstate Biotechnology, Lake Placid, NY) was used at a dilution of 1:1,000. After overnight incubation primary antibody was washed off in 1× PBST (1× PBS + 0.05% Tween 20) followed by the addition of secondary antibody (anti rabbit IgG-horseradish peroxidase conjugate, Amersham Bioscience, Piscataway, NJ) at a dilution of 1:2,000 in PBST for one hour at room temperature. The membranes were washed as before and visualized using enhanced-chemiluminescence reagents (Pierce) according to the manufacturer's protocol. Anti-α-Tubulin mouse antibody (Calbiochem, Cat#: CP06) was added at a dilution of 1:10,000 in PBST, and the secondary antibody (goat anti mouse IgG – horseradish peroxidase conjugate, PerkinElmer Life Sciences, Wellesley, MA) was added at a dilution of 1:5,000 in PBST for one hour at room temperature.

### Electrophoretic mobility shift assay (EMSA)

EMSA was performed using a DIG Gel shift Kit 2^nd ^generation (Roche applied science, Indianapolis, IN) essentially described in the manufacturer's protocol. Briefly, single-stranded oligonucleotides (oligo) 5'-GGC CTT TAT GTG CGC CGG AGA GAC CCG CCC-3' and its complementary oligo were synthesized (Invitrogen, San Diego, CA) and annealed to make double-stranded (ds) REST oligo. The core sequence is underlined. The oligo 5'-GGC CTT TAT GTG CGC TTT TGA GAC CCG CCC-3' and its complementary oligo were synthesized and annealed as mutant control. The ds oligos were terminally-labeled with non-radioactive DIG-11-ddUTP by terminal transferase and incubated with the protein extracts isolated from parental cells or cells transfected with REST/NRSF or mutant p73 expression vectors for 15 min at room temperature. In addition, 10× or 25× of wild-type unlabeled oligo were added to the labeled oligo for competition analysis. The samples were electrophoresed on a 6% DNA Retardation Gel (Invitrogen, CA) at 80 V for 1 h followed by alkaline transfer to positive-charged nylon membrane and chemiluminescence detection.

### Nuclei isolation and micrococcal nuclease (MNase) digestion

To partially digest cellular chromatin with MNase, a T75 flask of cells was harvested by trypsinization. The cells were washed once with Dulbecco's PBS, and then washed twice with 5 ml ice-cold tris buffered saline (TBS) (10 mM tris, pH 7.5, 150 mM NaCl, 5 mM MgCl_2_). The cells were then washed with 2 ml of ice-cold CB buffer (10 mM Tris, pH 8.0, 10 mM NaCl, 5 mM MgCl_2 _supplemented with 1 mM dithiothreitol on the day of use), and then washed again with 1 ml ice-cold CB. The cells were pelleted once more, and lysed by resuspending them in 0.5 ml ice-cold CB plus 0.5% Triton X-100. Nuclei were pelleted by centrifugation at 2000 × g for 5 min. The nuclei were resuspended in 50 μl ice-cold CB and 9 μl aliquots of nuclei were added to 0.5 ml microfuge tubes containing 1 μl MNase freshly diluted in MNase digestion buffer (20 mM PIPES, pH 7.0, 1 mM MgCl_2_, 10 mM NaCl, 250 mM sucrose, supplemented with 1 mM CaCl_2_, and 5 mM 2-mercaptoethanol). A stock solution of MNase (Fermentas Cat#: EN0181) was purchased and stored at -20°C. Nuclei were digested with different concentrations of MNase at room temperature for 20 min. After the MN reaction, the nuclei were digested by addition of 225 μl proteinase K solution (20 μg proteinase K/ml in 0.5% SDS, 10 mM tris, pH 8.0, 5 mM EDTA) followed by overnight incubation at 37°C. DNA was precipated at -20°C for 1 h after addition of 25 μl 3.0 M sodium acetate, pH 5.2, and 500 μl propanol. After centrifugation, DNA was washed with 70% ethanol prior to electrophoresis.

### Southern blot hybridization

The DNA was subjected to gel electrophoresis (1.2% agarose gel, 6 hours at 40 volts). After electrophoresis, the gel was treated twice with denaturing solution (0.4 N NaOH solution) for 15 min at room temperature. The DNA was transferred for 6 h from the gel to a positively charged nylon membrane using an alkaline transfer protocol. Hybridization was performed overnight at 42°C. To make a whole plasmid probe, linear pREP-SEAP DNA was labeled using Prime-a-Gene^® ^Labeling System (Cat. No: TB049) from Promega (Madison, WI) based on the random-primed method.

### Chemiluminescent SEAP assays

Promoter activity was analyzed by measuring SEAP reporter gene activity using GreatEscape kit according to the manufacturer's protocol (BD Biosciences). Transiently or stably transformed cells were plated in 60-mm plates at 5 × 10^5 ^cells/plate. After 2 days when an evenly distributed monolayer had formed, the medium was replaced with fresh DMEM medium and the flasks were incubated overnight. Fifteen μl of culture medium was collected and mixed with 45 μl of dilution buffer. The samples were incubated at 65°C for 30 min, after which 60 μl of assay buffer was added to the cooled samples. The reaction mix then was incubated at room temperature for 5 min followed by addition of 60 μl of 1.25 mM CSPD substrate according to the manufacturer's protocol. The chemiluminescent signal was measured at 420 nm by 20/20^n ^Luminometer (Turner Biosystems, Sunnyvale, CA) after 10 min incubation. Each construct was tested using a minimum of three replicates and the data were collected and normalized as SEAP units relative to the controls.

### Reverse transcriptase PCR (RT-PCR)

For RT-PCR, total RNA from cultured cells was isolated by Trizol reagent (Invitrogen). RT-PCRs were performed using Superscript One-Step RT-PCR (Invitrogen) with 0.5 μg of total RNA and two primer sets per reaction tube: one set for the actin as a control and another for the REST/NRSF. The RT-PCR primers were designed to bind in different exons to avoid unintentional amplification of potential genomic DNA contamination. Their sequences are as follows: Actin: 5'-ATT CCT ATG TGG GCG ACG AG-3' and 5'-TGG ATA GCA ACG TAC ATG GC-3'; REST/NRSF: 5'-TGT ATT TGA GGC ATC AGG TGC TC-3' and 5'-GTG TGG TGT TTC AGG TGT GCT G-3'; ICP4: 5'-CGA CAC GGA TCC ACG ACC C-3' and 5'-GAT CCC CCT CCC GCG CTT CGT CCG-3'; LAT: 5' CGG CGA CAT CCT CCC CCT AAG C 3' and 5' GAC AGA CGA ACG AAA CGT TCC G 3'; ICP0: 5'-TTC GGT CTC CGC CTG AGA GT-3' and 5'-GAC CCT CCA GCC GCA TAC GA-3'. The reverse transcription/PCR reaction was carried out at 45°C for 20 min followed by 25 cycles of 94°C for 30 s, 55°C for 30 s, and 68°C for 30 s. The RT-PCR products were analyzed by 2% agarose gel electrophoresis. The Kodak Gel-Logic 100 system was utilized for quantification.

### Chromatin Immunoprecipitation (ChIP) assays

The protocol was essentially described in Hsia and Shi [32] with modification according to the manufacturer's manual. Briefly, the cell monolayer was treated with 1% formaldehyde solution for 10 min at room temperature. The monolayer was then scraped into 15 ml tubes and subjected to the nuclei isolation protocol described previously. The samples were fragmented by MNase digestion (3.75 units/μl on ice for 1 h). The reaction was stopped by EDTA at the final concentration of 50 mM. The nuclei was pelleted and incubated with 400 μl of SDS lysis buffer (1% SDS, 10 mM EDTA, and 50 mM Tris-HCl {pH 8.1}) containing proteinase inhibitor (Complete Mini, Roche) on ice for 10 min. The lysed samples were spun for 10 min at 13,000 g with a refrigerated Eppendorf microfuge at 4°C, and the supernatant was diluted 10-fold with dilution buffer (25 ml; 0.01% SDS, 1.1% Triton X-100, 1.2 mM EDTA, 16.7 mM Tris-HCl {pH 8.1}, and 167 mM NaCl) containing protease inhibitor as described above. Immunoprecipitation was then performed with a ChIP assay kit essentially as described by the manufacturer with an antibody against acetylated H_4 _(Cat#: 17-229, Upstate Biotechnology, Lake Placid, N.Y.). This is a polyclonal antibody generated by using peptide corresponding to amino acids 2–19 of Tetrahymena histone H4 [AGG_Ac_KGG_Ac_KGM G_Ac_KVGA_Ac_KRHSC], acetylated on lysines 5, 8, 12 and 16. For immunoprecipitation of FLAG fusion protein, EZview™ Red ANTI-FLAG^® ^M2 Affinity Gel (Sigma-Aldrich Biotechnology, St. Louis, MO, Cat#: F2426) was utilized. Anti-CoREST, against human CoREST corresponding to residue 109–293, was purchased from Upstate Biotechnology (Cat#: 07-455). To analyze the DNA immuno-precipitated by the antibody or affinity gel, PCR amplification was performed on the precipitated DNA with primers (5'-TGG GGT GGG CGG GTC TTT C-3' and 5'-ACG AAC GAC GGG AGC GGC TG-3') against HSV-1 RE-1/NRSE. The primer sequences against hygromycin B gene (HGB) are 5'-TTG TTG GAG CCG AAA TCC G-3' and 5'-CAA ACT GTG ATG GAC GAC ACC G-3'.

For each reaction, a 50-μl, 25-cycle PCR was carried out in the presence of 10 pmol of the primers. Each cycle consisted of 1 min at 94°C, 40 s at 50°C, and 1 min at 72°C. Each experiment was done at least twice with similar results. The ChIP PCR products were analyzed by 2% agarose gel electrophoresis.

## Competing interests

The author(s) declare that they have no competing interests.

## Authors' contributions

RCP generated the reporter plasmids pICP4 and pICP22, performed the transient transfections, carried out the RT-PCR, performed the MNase digestion and Southern blot hybridization, generated the stable cell lines, and performed the EMSA, and ChIP. GRB assisted the preparation of reporter plasmids, performed the transfection experiments, confirmed the RT-PCR results, assisted the MNase digestion and Southern analysis, and maintained the stable cell lines. BG performed the Western blot analysis and repeated the transfection experiments. TCH participated in the identification of the RE-1/NRSE, designed the reporter plasmids, participated in the experimental strategy, and helped the manuscript preparation. JMH prepared the expression vectors, designed the experimental strategy, discussed the experimental data, conceived the strategic plan, and participated in the manuscript preparation. SVH initiated the project, identified the RE-1/NRSE, prepared the original reporter plasmids to make pICP4 and pICP22, performed the MNase digestion and Southern blot hybridization, directed all the experimental approaches, analyzed the preliminary data, supervised the work, and prepared the manuscript. All authors read and approved the final manuscript.
